# Pyorrhea Treatment, a New Feature

**Published:** 1906-01-15

**Authors:** W. V.-B. Ames


### PYORRHEA TREATMENT, A NEW FEATURE.

BY W. V.-B. AMES.

However much our opinions may differ as to the causation and modifying influences of the disorder we have come to call pyorrhea alveolaris, we are happily almost concerted as to the value of many methods of treatment. Any dissertation on the management of this lesion will be found prefaced by the inference that it is a forgeone conclusion that the surgical removal of foreign accretions is the matter of primary importance, and that ultimate good results depend upon the thoroughness with which this is accomplished. So, also, in any desperately bad case, presenting teeth seriously loose in their sockets, the importance of surgical rest is uniformly recognized, this being secured by proper adjustment of the occlusion and by the construction of sufficiently rigid splints. Apropos of this reference to splints, I will venture that the dental practice of the more or less near future will recognize extensive fixed bridging as a means of securing for a patient, for a gratifying period, the services of a natural denture, which the ravages of pyorrhea alveolaris to-day almost universally condemns to the forceps.

A feature of the treatment of pyorrhea, which it is my main purpose to present to-day, it seems ought to be as universally accepted as being logical and practicable as the removal of accretions, the adjustment of the occlusion, or the giving to loose teeth surgical rest. This feature of treatment I will announce in a general way to be "*continued mild medication.*" This medication may be the presenting of a condition which will give to the tissues continued mild stimulation, a continued mild astringent effect, or possibly in some stages a continued mild sedative effect; probably in all cases a continued mild sterilizing effect being called for.

The suggestion to me of what I offer which may savor of innovation has come from clinical observation of persons, some of whom I cannot mention by name, after the placing of bland and inoffensive material upon the roots of the teeth, which in time must be acted upon by the fluids of the environment to produce a trace of a potent medicinal agent, affording a continued mild medication.

Examples easily mentioned, which probably have suggested to me a line of medication which has proven efficacious, are, the placing of silver bands about the roots of teeth to furnish silver salts for their typical effect, the credit of which I cannot place, and the method suggested by Dr. E. R. Carpenter before this society of filling the spaces at the bifurcation of multi-rooted teeth with oxyphosphate of copper, when a space exists at that location from recession of tissues, getting in this instance a "continued mild medication" reasonably expected from the mass of this material in such an environment.

In observing such results, the query has presented: How can such effects be secured to an extent in a simple and easy manner about and within the pockets that a pyorrhea case presents?

As an example of simple continued medication, we have the employment of so-called milk of magnesia, which is theoretically and probably in reality an antacid of considerble value. Let us suppose that occasion calls for a continued antacid effect within a pyorrhea pocket, and suppose that a paste of magnesium hydrate, the sediment of milk of magnesia, were available, which could be readily introduced to the extreme depth of this pyorrhea pocket. Is it not reasonable to believe that some of this paste would remain in position for several days and an antacid state be maintained until the last trace has been removed by chemical action or otherwise?

Suppose, further, that a continued zinc salt effect be desired within such pocket. The zinc salt effect is astringent and to stimulate healthy granulations. Zinc hydrate, of itself a bland material, may be made into a paste and carried to the extreme depth of a pocket to exercise its typical action for a prolonged period because of the formation of soluble zinc salts by the action of acids developed *in situ* to say nothing of the effect of the hydrate *per se* from continuous contact. Zinc hydrate seems the material *par excellence* for the position, because of its potency and unobjectionable color. Ferric hydrate might be as efficacious but carries an objectionable color. A combination of magnesium and zinc hydrate might quite naturally suggest itself. These pastes would ordinarily be made with glycerine or glycerine and gelatin combined, and may be made to contain such sterilizing agents as are supposed to be valuable additions to tooth pastes and mouth washes. These pastes may be used by the patient in the sanitation of the mouth with reasonable anticipation of decided benefit from the lodgment of the hydrates beneath the gum margin and between the teeth. Such pastes may be introduced by the dentist by being placed beneath the gum margin and being carried farther by a massaging process. A cement syringe, such as that manufactured by Dr. Siqueland, of St. Paul, would introduce the paste well into the depths of the pocket, especially if the point of the ejector tube be flattened.

If a zinc hydrate paste be used at bedtime, there will be no evidence from taste, of a zinc compound, but on awakening hours after, the presence in the mouth of zinc salts is unmistakable. This may bring up the possibility of zinc poisoning, which, while not a persistently serious affliction, is a possibility worth consideration. Clinical data indicates that there need be little danger of untoward results if the zinc compound entered the stomach with or soon after a meal. In counting on the continued medication, there must be the possibility of traces of zinc salts entering the empty stomach with the saliva. In watching a few cases experimented upon I have not been able to detect annoying symptoms such as could be attributed to zinc salts or hydrate entering the alimentary canal. With exercising care to remove the paste from the papillose surface of the tongue by brushing or scraping, I feel that there need be little fear of zinc poisoning of even a susceptible subject. A serious mouth malady might even justify a possible temporary intestinal derangement.

In addition I have only to say that if I were in dental practice at present, I would rely upon this method of medication in all cases exhibiting pyorrhea or a pyorrheal tendency. I have not given any definite formulae, as the paste should be simply one of glycerine or glycerine and gelatine, loaded up with the hydrate and containing essential oils, etc., to suit your individual preferences. The zinc hydrate is easily obtained by precipitation by aqua ammonia of such a zinc salt as the sulphate.—*Review.*

